# Editorial: Understanding the Role of the Time Dimension in the Brain Information Processing

**DOI:** 10.3389/fpsyg.2017.00240

**Published:** 2017-02-23

**Authors:** Daya S. Gupta, Hugo Merchant

**Affiliations:** ^1^Department of Biology, Camden County CollegeBlackwood, NJ, USA; ^2^Department of Behavioral and Cognitive Neurobiology, Instituto de Neurobiología, UNAMQuerétaro, Mexico

**Keywords:** temporal processing of sensory information, neural oscillations, Schizophrenia, timing and time perception, sensory information processing

An accurate representation of time-dimension in the neuronal circuits is required for a successful interaction of the brain with the four-dimensional physical world. Time-dimension, unlike other three dimensions of our physical universe, is never perceived as a novelty, but only reported as the flow of time. As there are no known neurological or psychiatric disorders that are associated with the loss of the sense of flow of time, this suggests that the functions of the brain involve processing of temporal information (Merchant et al., 2013). Moreover, psychological flow of time is likely the result of the perception of the physical nature of the time-dimension.

The information about a stimulus coded by neural circuits can be understood in terms of Shannon information, which is the arrangement of spikes (an absence or presence) in time-bins of specific size along the time-dimension (Gupta and Chen, 2016a,b). Thus, Shannon information inherently incoporates time-bin as the time-dimension in information processing. Encoded stimulus characteristic, can be decoded or utilized in brain circuits by processing this information, referred as the temporal processing of information. Thus, it is implicit that the information processing, underlying various cognitive functions of the brain, is coupled with the invariant time-dimension.

Several novel findings are reported in this Special Issue, which bring us closer to understanding the role of the time-dimension in the brain information processing. These include the representation of the physical time in neural circuits, temporal processing of information, the role of prior information in the internal representation of rhythmic time, and neural oscillations in timing behavior and perception.

## Brain oscillations in timing behavior and perception

Brain oscillations are a key element on information processing and play a crucial role in the communication between and within different cortical and subcortical areas (Buzsáki, 2006). Neural oscillations have been linked to different high cognitive functions of which timing and time perception constitute one of the most studied (Treisman, 1963; Matell and Meck, 2004; Gupta, 2014; Kononowicz and van Rijn, 2014; Merchant et al., 2015a).

Kononowicz and van Wassenhove in this issue, review different oscillatory models that explain how the brain may subserve interval timing in millisecond range, but they focus mainly on the Striatal Beat Frequency (SBF) model and the new evidence that supports it. In contrast to other models, the SBF is more biologically plausible and implies the existence of cortical oscillators of various frequencies. At the onset of a timed interval, cortical oscillators are phase-reset and, at the offset of the interval, the state of these cortical oscillators is read by the medium spiny neurons of the striatum. Due to the fact that spiny neurons are coincidence detectors of the cortical input, the output of these striatal neurons could in principle tell time. Now, the authors suggest that the cortical input on the spiny neurons could be any stable neural pattern including neural avalanches (Crowe et al., 2014; Merchant et al., 2015b) or population state activity (Merchant et al., 2014; Mello et al., 2015). The authors also emphasize the notion that oscillation modulation for the SBF should be phase-reset at the beginning of a time interval, and that the frequency of the oscillation should be modulated by dopaminergic agents. None of these properties have been properly tested yet. In addition, this opinion letter underlines the fact that recent studies have shown that beta not only alpha oscillations are deeply involved in prediction of events during rhythmic tasks. Last, this paper suggests that the coherence between cortical and striatal signals is a fundamental element for generating a realistic model that includes both structures.

The paper by Chang et al. investigates the changes in beta oscillations induced by unpredicted changes in pitch, during an oddball EEG experiment where human subjects passively listened to isochronous auditory sequences with occasional unpredicted deviant pitches. These researchers tested the notion that if the induced beta power only reflects predictive timing, the occasional unpredicted pitch changes should not affect the ongoing beta entrainment behavior, given that the pitch deviants are presented at the predicted rhythmic time points. In contrast, their experimental results indicate that induced (non-phase locked) beta power was modulated by the unpredicted deviant pitches, suggesting that beta power is associated with predictive perceptual processing for the stimuli what (the pitch) and when (the tempo of the isochronous beat). The authors interpret these findings as the evidence that predictions for what and when are dynamically processed through attentional networks, and that beta oscillations in auditory cortex reflect the functional significance of sensory prediction and prediction error processes.

Kumar et al. report findings from a study of the McGurk effect, in which semantically-incongruent visual information modulates auditory perception. Authors employed incongruent audio-visual (AV) pair (audio/*pa*/ superimposed on the video of the face articulating /*ka*/) to induce the cross-modal percept/*ta*/. They find that for asynchronous AV (audio-visual) stimuli, a broadband enhancement, in the global coherence at theta, alpha, beta, and gamma bands, aids the cross-modal perception (percept/*ta*/). Long-range oscillations (alpha and beta bands) aid in the multidimensional processing of information of asynchronous AV stimuli by providing temporal window of integration, which is a specific phase of long-range oscillations (Gupta and Chen, 2016a). Asynchronous AV stimuli can be integrated in the same phase—temporal window of integration – of a long-range oscillation, which is possible due to the difference in the delays prior to their arrival at respective processing circuits (Gupta and Chen, 2016a). This difference in delays helps to eliminate the difference in the time of the initial presentation of asynchronous AV stimuli. In contrast to asynchronous AV stimuli, the processing of synchronous AV stimuli is temporally coupled to same coordinates on the time-axis, and therefore, it does not require the coupling to the same phase of a long-range oscillation to achieve simultaneous processing. Consistent with this argument, Kumar et al. observed desynchronization at alpha and beta- bands for AV stimuli. However, long-range oscillations may interfere with the integration of synchronous AV stimuli. This study highlights the important differences in the multisensory integration of synchronous and asynchronous AV stimuli.

The next paper by Chen and Huang studied alpha and beta modulations in a temporal version of n-back working memory task. Their findings reveal that while posterior alpha band reflects inhibition of task-irrelevant information, temporal region-distributed beta band activity is important for the active maintenance of temporal duration in the working memory.

Finally, Emmons et al. investigated the LFP oscillatory changes in the medial prefrontal cortex and the striatum during an interval timing task, where rats produced a 3 or 12 s interval. The results showed significant changes in delta and/or theta bands in the two areas during the following epochs of the task: after the cue that signaled the beginning of the time interval, throughout the interval, prior to the response that define the end of the interval, and after reward delivery. These findings support the notion that oscillatory activity between both areas of the motor cortico-basal-ganglia-thalamo-cortical circuit (Merchant et al., 2015a) is engaged in the temporal control of action in rodents.

## Mismatch negativity (MMN): represents mechanisms for extracting physical time information

MMN is an event-related potential (ERP) wave, which reflects neuronal processes underlying the brain's automatic reaction to novel or deviant as well as unattended sensory stimuli (Näätanen et al., 2007). In a work submitted to this Research Topic, Wang et al. extracted and correlated several different parameters—temporal parameters (onset, offset, and peak latency) and wave shape parameters (amplitude, average amplitude, upslope, downslope)—characterizing the MMN waves produced by deviant sound. Their results revealed only one important correlation: a positive correlation between the MMN amplitude and the slope of decaying phase, also called downslope (Wang et al.). The authors argue that this represents an efficient feedback process, which allows MMN to return to the baseline within a predefined time-window. This also suggests a coupling between the neuronal processes associated with deviant stimuli and a representation of the physical time-axis in the brain. This coupling may subserve the mechanism to input the physical time information into brain circuits, which would calibrate endogenous oscillators in a distributed modular clock model (Gupta, 2014).

In another study Schirmer et al., using MMN paradigm, deviant stimulus was created by subjecting one surprised and one neutrally spoken “Ah” to a speech manipulation procedure creating a 378 ms (short) and a 600 ms (long) exemplar. In both emotional conditions, short or long exemplars were used as standard or deviant stimuli. When short exemplar served as the standard, long exemplar was used as the deviant stimulus, and the vice versa. Authors observed a MMN-like negativity—climbing negativity that plateaued. Greater negativity for deviants than standards emerged shortly after the deviant onset, before the standard or deviant duration had lapsed. This suggested that listeners implicitly tracked sound speed and detected speed changes (Schirmer et al.). Continuous detection of the changes in the speed of unattended sounds would play a role in the calibration of endogenous oscillators in modular clock mechanisms (Gupta, 2014), including those that play a role in speech production.

## Simultaneity judgment of temporal events

The paper by Yarrow et al. tested different paradigms to optimally determine the relative judgment of two or more simultaneous events. Specifically, they argue that the dual presentation simultaneity judgment (2 x SJ) task is the most desirable. In this tasks subjects are asked to discriminate which of two pairs of stimuli presented consecutively was the most synchronous. They develop an appropriate signal detection theory model to analyze the 2xSJ data, and finally, they compare the data from the novel task with more conventional simultaneity tasks. Compared to classical tasks such as the temporal order judgment task, the 2 x SJ provides more constrained estimates of sensory noise, which indicates a more straightforward decision process. In fact, the 2 x SJ requires explicitly to decide which alternative timing relationship is most synchronous on any given trial, rather than revealing what range of relationships are perceived as synchronous. Consequently, 2 x SJ will serve as a crucial complement to existing methods for investigating subjective timing.

## Monkeys and humans share the ability to internally maintain a temporal rhythm

García-Garibay et al. demonstrate the ability of the rhesus monkeys and humans to perceive and maintain rhythms of different pace in the absence of sensory cues or motor actions. They use a visuospatial task in which subjects observe and then internally track a visual stimulus that periodically changed its location along a circular path. The proportion of trials in which subjects correctly estimated the position of the stimulus, along with other variables were determined in this study. Both species showed variability, consistent with Weber Law, where time independent variability increased as a function of timed duration (Zarco et al., 2009). In a different version of this task tested in humans, which reveals patterns of timing errors, shows that subjects tend to lag in fast rhythms and to get ahead in slow ones. The authors argue that a mean tempo might be incorporated as prior information, helping to reduce the effect of noise in time estimation and production tasks (García-Garibay et al.).

## Abnormal temporal processing of information: in schizophrenia and psilocybin-induced states

A meta-analysis of functional MRI studies in schizophrenia, comparing brain structures, activated or inactivated by time perception task and increasing levels of cognitive difficulty, revealed bilateral overlapping of cortical and subcortical regions, particularly frontal areas (mainly right BA 6), as well as parietal regions and the basal ganglia (Alústiza et al.). The overlapping regions, which are primarily in the right hemisphere, showed reduced rather than increased activity in schizophrenic patients relative to control subjects, not only by time perception tasks but also by an increase in the level of difficulty of non-temporal tasks (Alústiza et al.). Reduced activity of various brain structures is consistent with the prevailing view that there is an impaired functional connectivity of brain regions in schizophrenia (Hutchison et al., 2013). Thus, dysconnectivity affects common networks in schizophrenia, which are engaged by both the increasing task difficulty and time perception tasks.

In a commentary, Shebloski and Broadway discuss a paper by Wittmann et al. (2007). The study by Wittmann et al. (2007) showed a decreased ability to accurately produce intervals longer than 3 s and synchronize finger-tapping to auditory beats separated by more than 2 s under the influence of psilocybin (Wittmann et al., 2007). The effects on timing performance were accompanied by working-memory deficits and subjective changes in conscious state. Shebloski and Broadway also noted that schizophrenia, which is associated with similar changes in subjective state, such as hallucinations, is also associated with timing deficits in sub- and supra-second range. They further point out that slowing of perceived time induced by psilocybin and schizophrenia may share certain common mechanisms, such as 5-HT2*A* receptor activities. Thus, Shebloski and Broadway propose that commonalities across pharmacological treatments and psychiatric disorders should be explored within a common experimental paradigm.

It should be also noted that in contrast to the effects of psilocybin administration, which are pharmacological, the pathophysiology underlying schizophrenia involves defects at many levels, such as circuit, molecular and morphological levels. Therefore, to interpret the results of experiments in terms of underlying pathophysiology will involve many challenges.

## Study of modular interactions between brain regions using artificial systems

Maniadakis and Trahanias test a model of artificial cognitive system, which has the ability to sense when events have occurred and how long they have lasted. Authors employ a set of neural networks in their model, to synthesize modules, similar to the modular parts of the human brain. Inspired by the striatal beat frequency model of interval timing (Matell and Meck, 2004; Meck et al., 2008), authors incorporated a module in their artificial system, which transforms oscillatory inputs into a composite time flow representation.

Authors used a coevolutionary scheme (Maniadakis and Trahanias, 2008) to train the model, and improve the collaboration between component neural networks, forming modules. The coevolutionary procedure, after 500 generations, produced a modular system that memorizes the duration and time of occurrence of events. Such methods can be a useful computational tool for the study of modular interactions in brain networks.

Various papers in this special issue describe that modulations of beta-range oscillations play an important role in the timing behavior. Beta power increased as working memory load increased in a temporal version of n-back working memory task (Chen and Huang), which suggests that beta oscillations play an important role in the functioning of brain networks serving the levels of cognitive effort and time perception that is likely affected in schizophrenia (Alustiza et al.) Future studies should look more closely at the role of the representation of the time-dimension in the temporal processing of information in the brain, which may be affected in psychiatric illnesses.

## Author contributions

All authors listed, have made substantial, direct and intellectual contribution to the work, and approved it for publication.

### Conflict of interest statement

The authors declare that the research was conducted in the absence of any commercial or financial relationships that could be construed as a potential conflict of interest.
